# Geriatric Nutritional Risk Index Is Associated with Unique Health Conditions and Clinical Outcomes in Chronic Kidney Disease Patients

**DOI:** 10.3390/nu11112769

**Published:** 2019-11-14

**Authors:** Ting-Yun Lin, Szu-Chun Hung

**Affiliations:** Division of Nephrology, Taipei Tzu Chi Hospital, Buddhist Tzu Chi Medical Foundation, and School of Medicine, Tzu Chi University, Hualien 970, Taiwan; water_h2o_6@hotmail.com

**Keywords:** cardiovascular disease, chronic kidney disease, geriatric nutritional risk index, mortality, nutrition screening

## Abstract

Malnutrition is prevalent in patients with chronic kidney disease (CKD). However, current nutrition screening tools are not specific to the CKD population. In the present study, we aimed to investigate whether the geriatric nutritional risk index (GNRI), a simple tool designed for assessing nutrition-related risks in the elderly population, is associated with unique aspects of CKD such as fluid status, residual renal function, proteinuria, and inflammation, and whether it predicts clinical outcomes. The GNRI was calculated by incorporating serum albumin and anthropometric measurements in 326 patients with nondialysis stage 3–5 CKD who were followed up from September 2011 to March 2017 for end-stage renal disease (ESRD) and the composite outcome of all-cause death and cardiovascular events. Patients were stratified into tertiles according to baseline GNRI levels. Patients in the lowest GNRI tertile were more likely to have significantly higher levels of overhydration, proteinuria, and serum inflammatory markers and tended to have lower lean body mass and estimated glomerular filtration rate when compared with patients in the middle and upper GNRI tertiles. In multivariate linear regression analyses, the GNRI was independently associated with overhydration, proteinuria, and interleukin-6. During a median follow-up of 4.9 years, 101 patients developed ESRD; 40 deaths, and 68 cardiovascular events occurred. Patients in the lowest GNRI tertile had significantly increased risks of ESRD (hazard ratio (HR): 3.15, 95% confidence interval (CI): 1.95–5.07, *p* < 0.001) and the composite outcome (HR: 1.79, 95% CI: 1.10–2.92, *p* = 0.019) in fully adjusted models (reference: middle and upper GNRI tertiles). The GNRI takes CKD-specific health conditions into account. In addition, CKD patients with lower GNRI scores had a significantly higher risk of adverse clinical outcomes. Our findings suggest that the GNRI is an appropriate tool for nutrition screening and a prognostic predictor among patients with nondialysis stage 3–5 CKD.

## 1. Introduction

The burden of chronic kidney disease (CKD) continues to increase [1]. Protein energy wasting (PEW) is one of the most prevalent complications and a significant contributor to adverse outcomes in this population [2,3]. Therefore, nutritional management is of paramount importance for patients with CKD. The essential approach to nutritional management is the early identification of CKD patients who are at risk for PEW by nutrition screening. A wide variety of screening tools are available including the Mini Nutritional Assessment (MNA) [4], Malnutrition Screening Tool (MST) [5], Malnutrition Universal Screening Tool (MUST) [6], and Nutritional Risk Screening-2002 (NRS-2002) [7]. However, there is currently no specific or validated screening tool available for CKD patients.

Although these screening tools are quick and easy to use, all require a brief patient interview. Questions that are common to these screening tools include asking about involuntary body weight changes and the amount of oral intake. Responses to these questions depend on the patient’s ability to provide accurate data, which might be troublesome in elderly CKD patients or those with cognitive impairment. In addition, patients with CKD have unique metabolic and nutritional abnormalities. Proper evaluation of the nutritional status of these individuals by nutrition screening must take into account the influences of CKD such as the patient’s fluid status, residual renal function, proteinuria, inflammation, and renal replacement therapy modality [8].

The geriatric nutritional risk index (GNRI), a simple objective index of malnutrition, is used to estimate the prognosis of elderly patients [9]. The GNRI is calculated from the serum albumin level and the ratio between the actual and ideal body weight, which are two important diagnostic criteria for PEW as defined by the International Society of Renal Nutrition and Metabolism (ISRNM) [10]. The GNRI is considered to be the simplest and most accurate in identifying hemodialysis patients with malnutrition among the various nutrition screening tools [11,12]. In the present study, we aimed to determine whether the GNRI is associated with CKD-specific health conditions and can predict clinical outcomes in patients with nondialysis CKD.

## 2. Materials and Methods

### 2.1. Study Population

The study design and participants in this prospective cohort study have been reported previously in detail [13]. Briefly, 395 prevalent patients aged ≥ 20 years with nondialysis CKD (defined as an estimated glomerular filtration rate (eGFR) <60 mL/min per 1.73 m^2^ calculated according to the Modification of Diet in Renal Disease formula) were assessed for eligibility for inclusion between September 2011 and December 2012. The exclusion criteria included active malignancy, liver cirrhosis, patients with cardiac pacemakers or metallic implants, patients who were amputees, patients who were pregnant, and patients with an acute cardiovascular (CV) event within the three months before the screening. Cardiovascular disease (CVD) was defined as coronary artery disease, as documented on coronary angiography or a history of myocardial infarction, NYHA class III to IV congestive heart failure, or stroke. The presence of diabetes mellitus (DM) was based on the current or past use of insulin and/or oral hypoglycemic agents. Hypertension was defined as either a blood pressure ≥ 140/90 mmHg or by current treatment with antihypertensive agents. All participants received a comprehensive CKD education program and were followed up every three months. We adhered to the ethical principles of the Declaration of Helsinki and obtained approval from the Institutional Review Board of Taipei Tzu Chi Hospital (01-XD13-034). Each patient provided written informed consent before participation.

### 2.2. Body Composition Measurements

Body composition was evaluated using a portable whole body bioimpedance spectroscopy device, the Body Composition Monitor (BCM, Fresenius Medical Care, Bad Homburg, Germany). The use of the BCM has been validated among healthy controls from the same ethnic background as the study population [13,14]. The BCM measures body composition by analyzing the electrical responses at 50 frequencies between 5 and 1000 kHz. Based on a three-compartment model, the body composition is separated into three components: overhydration, lean tissue mass, and adipose tissue mass [15]. Overhydration is the difference between the amount of extracellular water (ECW) in tissue that is detected by the BCM and the amount of ECW in tissue that is predicted by using physiological models under normal (euvolemic) conditions. Overhydration values were further normalized to the ECW and expressed as a percentage of the ECW. “Fluid overload” was defined as an overhydration value ≥ 7%, corresponding to the value of the 90th percentile for the reference cohort when the fluid status was measured with the same technology. Lean tissue mass and adipose tissue mass were normalized to the height squared and expressed as the LTI (lean tissue mass/height^2^) and FTI (adipose tissue mass/height^2^), respectively. 

### 2.3. Laboratory Measurements

All blood samples were collected in the morning after the patients had fasted overnight. The serum albumin concentration was measured by a bromocresol purple assay. The plasma levels of interleukin-6 (IL-6) and tumor necrosis factor-α (TNF-α) were determined with commercial ELISA kits, according to the manufacturer’s instructions (R & D Systems, Minneapolis, MN, USA). Proteinuria, as defined by the urine protein-to-creatinine ratio (UPCR), was estimated using the first morning urine specimen.

### 2.4. Geriatric Nutritional Risk Index

The GNRI was calculated as (14.89 + albumin (g/dL)) + (41.7 × body weight/ideal body weight) [9]. The ideal body weight was defined as the value calculated from the height and a BMI of 22 kg/m^2^ instead of the value calculated using the Lorentz formula in the original GNRI equation because of its validity [12].

### 2.5. Outcomes

The primary endpoint was end-stage renal disease (ESRD), defined as the need for chronic dialysis treatment or preemptive renal transplantation. The secondary endpoint was the time to the composite of death from any cause and CV events. The causes of death were ascertained from the official death certificates. CV events included nonfatal myocardial infarction, congestive heart failure, stroke, hospitalization for myocardial ischemia, or CV death. A trained physician who had no knowledge of the results of the GNRI independently reviewed all suspected CV events by examining each medical chart. The follow-up time for each participant started at the first study visit, when the GNRI was assessed. Patients were censored at the time of their last contact or the end of follow-up in March 2017.

### 2.6. Statistical Analyses

Categorical data are expressed as frequencies and percentages and were compared by the Chi-square and Bonferroni post hoc tests. Continuous data with or without a normal distribution are expressed as the means ± SDs or medians and interquartile ranges and were compared by one-way ANOVA or the Kruskal–Wallis test, followed by Tukey’s and Dunn’s post hoc tests, respectively. The associations of clinically relevant variables with the GNRI value were assessed using the Pearson’s correlation and univariate and multivariate linear regression models. Kaplan–Meier curves were constructed to examine the time to the outcomes for each tertile of the GNRI and were compared using a log-rank test. Cox proportional hazard models were used to estimate the hazard ratios (HRs) of the ESRD and the composite of death and CV events associated with the tertiles of the GNRI. Variables that were clinically relevant were used to adjust the multivariate models. The number of selected variables was restricted to no more than 1 covariate per 10 outcome events to avoid overfitting. A two-tailed P-value less than 0.05 was considered statistically significant. The statistical analyses were performed using the computer software SPSS, version 20.0 (SPSS Inc., Chicago, IL, USA).

## 3. Results

### 3.1. Patient Characteristics

**Figure 1:**
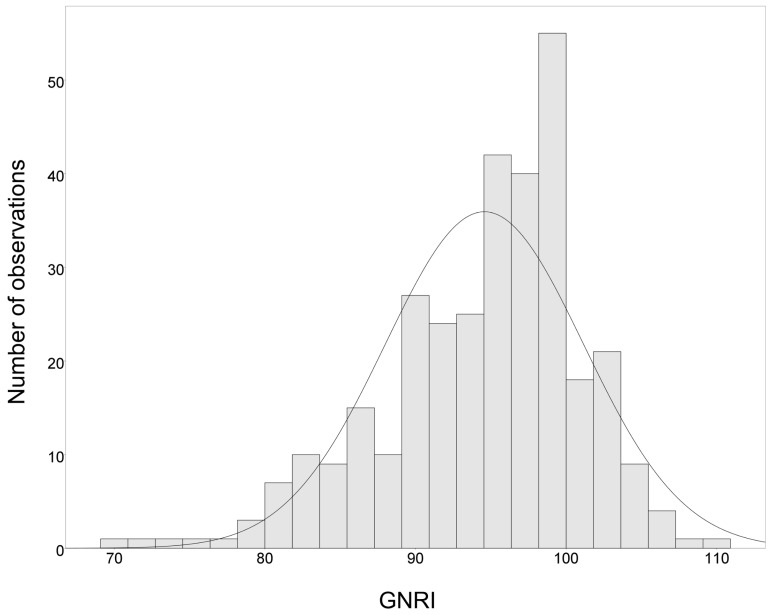
Distribution of GNRI. GNRI, geriatric nutritional risk index.

A total of 326 patients (102 women and 224 men) were enrolled and included in this analysis. The mean age of the participants was 66 ± 13 years; 45.4% (*n* = 148) had DM, and 23.6% (*n* = 77) had CVD. All participants had moderate to advanced CKD (mean eGFR 28.8 ± 14.7 mL/min/1.73 m^2^; 44.8%, 32.8%, and 22.4% had CKD stages 3, 4, and 5, respectively). Figure 1 shows the distribution of the GNRI scores in this study. The median (interquartile range) GNRI value was 95.4 (90.8–99.6). The patients were stratified into tertiles of GNRI levels as follows: tertile 1 (T1): GNRI 69.7–92.4, T2: GNRI 92.5–98.2, and T3: GNRI 98.3–110.2. Baseline patient characteristics stratified according to GNRI tertiles are summarized in Table 1. Patients in the highest tertile of the GNRI (T3) were more often male and had a significantly higher LTI and eGFR levels than those in the T1 and T2. In contrast, patients in the lowest tertile of the GNRI (T1) were more likely to be diabetic and have a significantly higher levels of overhydration, UPCR, systolic blood pressure, and plasma IL-6 and TNF-α concentrations compared with those in the other two tertiles (T2 and T3). Notably, there was no difference in age, the prevalence of CVD, the use of statins or renin-angiotensin-aldosterone system inhibitors, BMI, and FTI among the GNRI tertiles. The serum albumin concentration was incrementally higher with increasing tertiles of GNRI, whereas overhydration, UPCR, and plasma TNF-α levels were progressively lower.

### 3.2. Variables Associated with the GNRI

**Figure 2:**
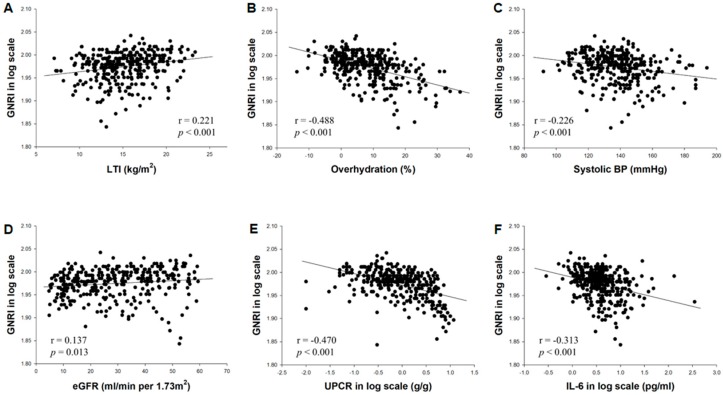
Correlations between the GNRI and baseline variables including LTI (**A**), overhydration (**B**), systolic blood pressure (**C**), eGFR (**D**), proteinuria (**E**), and IL-6 (**F**). Abbreviations: eGFR, estimated glomerular filtration rate; GNRI, geriatric nutritional risk index; IL-6, interleukin-6; ln, natural logarithm; LTI, lean tissue index.

The GNRI value was significantly correlated with several baseline variables (Figure 2) including the LTI (*r* = 0.221, *p* < 0.001), overhydration (*r* = −0.488, *p* < 0.001), systolic blood pressure (*r* = −0.226, *p* < 0.001), eGFR (*r* = 0.137, *p* = 0.013), proteinuria (*r* = −0.470, *p* < 0.001), and IL-6 (*r* = −0.313, *p* < 0.001). In a univariate linear regression model, male sex, the LTI, and the eGFR were positively correlated with the GNRI, whereas patients with DM and higher levels of overhydration, systolic blood pressure, proteinuria, and IL-6 had lower scores of the GNRI (Table 2). The GNRI remained significantly and negatively correlated with overhydration, proteinuria, and IL-6 in the multivariate model.

### 3.3. Association of the GNRI with Clinical Outcomes

During a median follow-up time of 4.9 years (3.0–5.3), 101 participants (31.0%) developed ESRD. The following CV events occurred in 68 patients (20.9%): hospitalization for myocardial ischemia (*n* = 13); fatal and nonfatal myocardial infarction (n = 14); congestive heart failure (*n* = 29); stroke (*n* = 5); and sudden cardiac death (*n* = 7). A total of 40 patients (12.3%) died including 23 deaths from non-CV causes and 17 deaths due to CV events. The most common causes of non-CV death were infections (*n* = 7), malignancies (*n* = 4), and gastrointestinal bleeding (*n* = 4).

**Figure 3:**
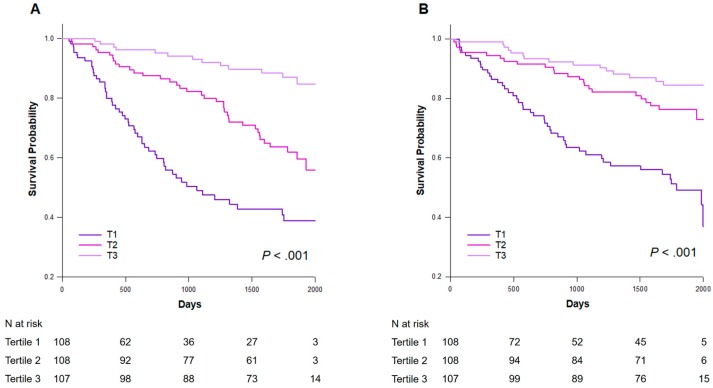
Kaplan–Meier survival curves for the adverse outcomes according to the GNRI tertiles. (**A**) ESRD (**B**) Composite outcome. T1: GNRI 69.7–92.4, T2: 92.5–98.2, T3: 98.3–110.2. GNRI, geriatric nutritional risk index; ESRD, end-stage renal disease.

In the Kaplan–Meier analysis, the ESRD rates were 46.8%, 33.9%, and 12.0% for patients in T1, T2, and T3, respectively (log-rank *p* < 0.001) (Figure 3A). The Kaplan–Meier curve demonstrated that patients in T1 had the highest risk of the composite outcome among the three groups (log-rank *p* < 0.001) (Figure 3B). As such, subsequent analyses modeled the GNRI as a dichotomous variable by applying a threshold of T1 vs. T2 + T3. In the Cox proportional hazard models, patients in T1 had an increased risk of ESRD after adjusting for age, sex, DM, systolic blood pressure, eGFR, proteinuria, overhydration, and IL-6 (HR: 3.15, 95% CI: 1.95–5.07, *p* < 0.001) (Table 3). In addition, T1 predicted an increased risk of the composite outcome in the fully adjusted models (HR: 1.79, 95% CI: 1.10–2.92, *p* = 0.019).

## 4. Discussion

In the present study, we examined the correlations between the GNRI and fluid status, residual renal function, proteinuria, and inflammation in nondialysis CKD patients. We found that the GNRI was associated with these unique CKD health conditions. Moreover, a lower GNRI score predicted a significantly higher risk of adverse clinical outcomes. We propose that the GNRI can be used as a CKD-specific nutrition screening tool.

Screening for malnutrition is the first step in nutritional management. Nutrition screening performed routinely will identify CKD patients at risk for PEW and ensure that further nutritional assessment and care is timely and appropriate. The most striking finding of the present study was that the GNRI was correlated with several major risk factors for accelerated CKD progression and CVD including DM, hypertension, eGFR, UPCR, and inflammatory markers [16,17]. Interestingly, we observed a significant and inverse relationship between the GNRI and overhydration, which has also been reported to be independently predictive of an increased risk of adverse renal and CV outcomes in nondialysis CKD patients [14,18]. The underlying mechanisms linking the nutritional status, overhydration, and proteinuria are not clear. Urinary protein loss may be related in part to undernutrition. However, proteinuria may merely reflect the degree of systemic inflammation and endothelial dysfunction [19]. One possible explanation for the association between overhydration and nutritional status is that overhydration can lead to gastrointestinal edema and inadequate nutrient intake [20]. Overhydration may also alter the colonic ecosystem (dysbiosis), which in turn disrupts the intestinal barrier and results in subsequent immune derangements and systemic inflammation [21]. Taken together, it appears that the GNRI incorporates situations unique to CKD and is a suitable nutrition screening tool for patients with CKD.

In the present study, we found that patients with lower GNRI scores had lower serum albumin concentrations and lean body mass and higher levels of inflammatory markers including CRP, TNF-α, and IL-6. Furthermore, the plasma IL-6 level was independently and inversely associated with the GNRI value. While the pathogenesis of PEW is multifactorial, systemic inflammation is regarded as an important contributing factor. Stenvinkel et al. reported a strong association between inflammation and malnutrition in advanced CKD, where the malnourished patients defined by SGA had significantly elevated CRP and fibrinogen levels, indicating an ongoing inflammatory process [22]. The activation of proinflammatory cytokines overcomes the adaptive response, protecting muscle, and reducing resting energy expenditure during insufficient protein and energy intakes, and induces muscle insulin resistance and subsequently protein catabolism, resulting in muscle loss [23]. Both elevated CRP levels and hypoalbuminemia have been shown to independently predict an elevated risk of all-cause mortality in patients with CKD stages 3 and 4 [24]. Notably, the plasma IL-6 concentration seems to be the most reliable predictor for adverse clinical outcomes among the inflammatory parameters. Barreto et al. demonstrated that the predictability of plasma IL-6 for overall and CV mortality in different stages of CKD was greater than that of other inflammatory parameters such as CRP, TNF-α, and albumin [25]. Honda et al. also reported that IL-6 was the only biomarker that, in competition with the other biomarkers, could classify the presence of malnutrition and subsequent CVD and mortality in patients with ESRD [26]. Our results suggest that the GNRI, a simple nutrition screening tool, assesses not only the nutritional status but also the underlying inflammatory process in CKD patients.

Although the GNRI was originally designed to screen nutritional status and predict short-term mortality in hospitalized elderly patients [9], the predictive value of the GNRI for prognosis has also been demonstrated in other patient populations. Lower GNRI levels have been associated with higher mortality risk in patients undergoing chronic hemodialysis and in patients with peripheral arterial disease or congestive heart failure [11,27,28]. Despite its simplicity, the GNRI’s outcome predictability outweighed those of several nutritional indices [9]. In the present study, although we did not aim to compare the GNRI with other parameters with regard to predicting clinical outcomes, we also found that a lower GNRI score predicted an elevated risk of ESRD and the composite outcome of death and CV events, even after adjusting for DM, systolic blood pressure, baseline eGFR, overhydration, UPCR, and the IL-6 concentration.

The strengths of this study, in comparison with other previous reports [29,30], are that we included several body composition measurements and inflammatory parameters in the analysis. Furthermore, we used spot UPCR instead of the dipstick method for the estimation of proteinuria. However, several limitations of our study should be acknowledged. First, as is the case for any observational study, we were unable to establish the causality of the relationship between the GNRI and clinical outcomes. However, the value of the GNRI as an independent predictor can be established. Second, the observed associations between the GNRI and clinical outcomes were based on baseline values. However, the GNRI may decrease over time in individuals with CKD and reach a minimum value around the time of an event. Therefore, a higher baseline GNRI would only bias the study results further toward the null hypothesis. Last, it is not known whether the GNRI tertiles observed in this study fell within the distribution of the GNRI scores in other patient cohorts. We analyzed the GNRI in tertiles rather than an established cutoff because the optimal GNRI value for risk stratification in CKD has not been determined. However, the cutoff of 92.4 for GNRI in the current study was close to the most accurate cutoff of 91.2 to identify a malnourished hemodialysis patient according to the malnutrition-inflammation score [12,31].

## 5. Conclusions

In patients with nondialysis CKD, the GNRI was correlated with CKD-specific health conditions. Moreover, a lower GNRI score was associated with an increased risk of developing ESRD and the composite outcome of mortality and CV events. Our findings suggest that the GNRI is an appropriate tool for nutrition screening and a prognostic predictor among patients with moderate to severe CKD.

## Figures and Tables

**Table 1:** Characteristics of CKD patients according to GNRI tertiles.

Characteristics	GNRI Tertiles			*p* Value
	T1 (*n* = 109)	T2 (*n* = 109)	T3 (*n* = 108)	
Age (years)	66.7 ± 14.2	66.4 ± 12.4	64.3 ± 13.3	0.375
Male sex, *n* (%)	69 (63.3%)	66 (60.6%)	89 (82.4%) ^b,c^	0.001
Smoking history, *n* (%)	25 (22.9%)	21 (19.3%)	21 (19.4%)	0.752
DM, *n* (%)	65 (59.6%)	45 (41.3%) ^a^	38 (35.2%) ^c^	0.001
CVD, *n* (%)	30 (27.5%)	24 (22.0%)	23 (21.3%)	0.497
CHF, *n* (%)	12 (11%)	9 (8.3%)	6 (5.6%)	0.346
CAD, *n* (%)	15 (13.8%)	8 (7.3%)	15 (13.9%)	0.227
CVA, *n* (%)	12 (11%)	9 (8.3%)	4 (3.7%)	0.124
RAAS, *n* (%)	66 (60.6%)	63 (57.8%)	67 (62.0%)	0.811
CCB, *n* (%)	64 (58.7%)	53 (48.6%)	49 (45.4%)	0.122
Furosemide, *n* (%)	36 (33.3%)	19 (17.4%)	12 (11.1%)	<0.001
No. of antihypertensives	2.32 ± 1.32	1.92 ± 1.36	1.84 ± 1.38	0.020
Statin, *n* (%)	31 (28.4%)	26 (23.9%)	29 (26.9%)	0.738
BMI (kg/m^2^)	25.3 ± 4.6	26.0 ± 3.9	26.4 ± 3.7	0.160
FTI (kg/m^2^)	9.5 ± 4.4	10.2 ± 4.0	9.6 ± 4.5	0.403
LTI (kg/m^2^)	14.5 ± 3.2	15.0 ± 2.9	16.2 ± 3.3 ^b,c^	<0.001
Overhydration (%)	13.2 ± 9.5	7.0 ± 7.2 ^a^	4.4 ± 6.4 ^b,c^	<0.001
Fat percentage (%)	27.1 ± 9.9	28.4 ± 8.7	26.1 ± 9.7	0.201
Systolic BP (mmHg)	142.3 ± 17.6	136.8 ± 18.6 ^a^	133.7 ± 13.9 ^c^	0.001
eGFR (ml/min/1.73 m^2^)	25.7 ± 14.6	27.2 ± 14.3	33.7 ± 14.2 ^b,c^	<0.001
UPCR (g/g)	2.40 (0.86–4.97)	0.84 (0.40–1.68) ^a^	0.38 (0.15–0.94) ^b,c^	<0.001
Albumin (g/dL)	3.1 ± 0.3	3.7 ± 0.1 ^a^	4.0 ± 0.2 ^b,c^	<0.001
Fasting glucose (mg/dL)	127 ± 46	118 ± 39	117 ± 39	0.147
Total cholesterol (mg/dL)	183 ± 47	175 ± 39	167 ± 33 ^c^	0.020
Triglycerides (mg/dL)	152 ± 107	171 ± 126	167 ± 109	0.441
hs-CRP (mg/L)	5.5 (1.7–12.3)	3.4 (1.1–9.1)	3.4 (1.2–8.0) ^c^	0.033
IL-6 (pg/mL)	5.00 (3.14–8.94)	3.17 (2.07–5.41) ^a^	2.93 (1.45–4.30) ^c^	<0.001
TNF-α (pg/mL)	8.51 (6.48–11.03)	6.15 (4.72–8.97) ^a^	5.48 (3.21–7.62) ^b,c^	<0.001

Abbreviations: BMI, body mass index; BP, blood pressure; CAD, coronary artery disease; CHF, congestive heart failure; CCB, calcium channel blocker; CKD, chronic kidney disease; CVA, cerebrovascular accident; CVD, cardiovascular disease; DM, diabetes mellitus; eGFR, estimated glomerular filtration rate; FTI, fat tissue index; GNRI, geriatric nutritional risk index; hs-CRP, high-sensitivity C-reactive protein; IL-6, interleukin-6; LTI, lean tissue index; No., number; RAASi, renin-angiotensin-aldosterone system inhibitors; T1, tertile 1; T2, tertile 2, T3, tertile 3; TNF-α, tumor necrosis factor-α; UPCR, urine protein creatinine ratio. GNRI levels: T1, 69.7–92.4; T2, 92.5–98.2; T3, 98.3–110.2. ^a^ T1 and T2 were significantly different (*p* < 0.05). ^b^ T2 and T3 were significantly different (*p* < 0.05). ^c^ T1 and T3 were significantly different (*p* < 0.05).

**Table 2:** Univariate and multivariate associations with the GNRI.

Characteristic	Univariate		Multivariate ^a^	
	β Coefficient (95% CI)	*p* Value	β Coefficient (95% CI)	*p* Value
Age	−0.035 (−0.089, 0.019)	0.204	-	-
Male sex	2.142 (0.612, 3.673)	0.006	-	-
DM (Presence)	−3.217 (−4.615, −1.818)	<0.001	-	-
Previous CVD (Presence)	−1.074 (−2.760, 0.613)	0.211	-	-
LTI (kg/m^2^)	0.466 (0.247, 0.684)	<0.001	-	-
Overhydration (%)	−0.373 (−0.446, −0.301)	<0.001	−0.245 (−0.322, −0.169)	<0.001
Systolic BP (mmHg)	−0.088 (−0.128, −0.047)	<0.001	-	-
eGFR (ml/min/1.73 m^2^)	0.068 (0.020, 0.117)	0.006	-	-
log UPCR (g/g)	−5.303 (−6.400, −4.207)	<0.001	−3.424 (−4.532, −2.316)	<0.001
log IL−6 (pg/mL)	−5.349 (−7.103, −3.596)	<0.001	−3.002 (−4.551, −1.458)	<0.001

Abbreviations: BP, blood pressure; CI, confidence interval; CVD, cardiovascular disease; DM, diabetes mellitus; eGFR, estimated glomerular filtration rate; GNRI, geriatric nutritional risk index; IL-6, interleukin-6; LTI, lean tissue index; UPCR, urine protein creatinine ratio. ^a^ Stepwise regression model.

**Table 3:** Multivariate Cox proportional hazard analysis for the relative risk of ESRD and the composite outcome calculated for the GNRI tertiles.

	ESRD		Composite Outcome	
	HR (95% CI)	*p* Value	HR (95% CI)	*p* Value
**Unadjusted**				
T2 + T3	Reference		Reference	
T1	3.57 (2.40–5.30)	<0.001	3.43 (2.24–5.26)	<0.001
**Model 1**				
T2 + T3	Reference		Reference	
T1	3.54 (2.38–5.25)	<0.001	3.08 (2.01–4.72)	<0.001
**Model 2**				
T2 + T3	Reference		Reference	
T1	3.15 (1.95–5.07)	<0.001	1.79 (1.10–2.92)	0.019

Abbreviations: CI, confidence interval; GNRI, geriatric nutritional risk index; HR, hazard ratio; T1, tertile 1; T2, tertile 2, T3, tertile 3. Model 1 was adjusted for age and sex. Model 2 was adjusted for the Model 1 variables and for diabetes mellitus, systolic blood pressure, estimated glomerular filtration rate, urine protein creatinine ratio, overhydration, and interleukin-6.
